# Disulfiram (Antabuse) Activates ROS-Dependent ER Stress and Apoptosis in Oral Cavity Squamous Cell Carcinoma

**DOI:** 10.3390/jcm8050611

**Published:** 2019-05-06

**Authors:** Priyanka Shah O’Brien, Yue Xi, Justin R. Miller, Amy L. Brownell, Qinghua Zeng, George H. Yoo, Danielle M. Garshott, Matthew B. O’Brien, Anthony E. Galinato, Peter Cai, Neha Narula, Michael U. Callaghan, Randal J. Kaufman, Andrew M. Fribley

**Affiliations:** 1 Department of Otolaryngology–Head and Neck Surgery, Wayne State University School of Medicine, Detroit, MI 48201, USA; shahpriyanka.a@gmail.com (P.S.O.); gyoo@med.wayne.edu (G.H.Y.); 2 Carman and Ann Adams Department of Pediatrics, Wayne State University School of Medicine, Detroit, MI 48201, USA; fc3128@wayne.edu (Y.X.); jrmiller816@gmail.com (J.R.M.); abrownel@med.wayne.edu (A.L.B.); dgarshot@ucsd.edu (D.M.G.); mcallagh@med.wayne.edu (M.U.C.); 3 Division of Hematology/Oncology, Department of Medicine, University of Alabama at Birmingham, Birmingham, AL 35294, USA; zengnwu@yahoo.com; 4 Wayne State University School of Medicine, Detroit, MI 48201, USA; caiy@umich.edu (P.C.); nnarula@stanford.edu (N.N.); 5 Henry Ford Hospital, Diagnostic Radiology Residency, Detroit, MI 48202, USA; bowser001@gmail.com (M.B.O.); anthony.galinato@gmail.com (A.E.G.); 6 Degenerative Diseases Program, Sanford Burnham Prebys Medical Discovery Institute, La Jolla, CA 92037, USA; 7 Molecular Therapeutics Program, Karmanos Cancer Institute, Wayne State University School of Medicine, Detroit, MI 48201, USA

**Keywords:** HNSCC, head and neck cancer, high throughput screen, HTS, Antabuse, disulfiram, NSC-1771, ROS, unfolded protein response, ER stress, OSCC, oral cancer

## Abstract

A paucity of advances in the development of novel therapeutic agents for squamous cell carcinomas of the head and neck, oral cavity (OSCC) and oropharynx, has stagnated disease free survival rates over the past two decades. Although immunotherapies targeted against checkpoint inhibitors such as PD-1 or CTLA-4 are just now entering the clinic for late stage disease with regularity the median improvement in overall survival is only about three months. There is an urgent unmet clinical need to identify new therapies that can be used alone or in combination with current approaches to increase survival by more than a few months. Activation of the apoptotic arm of the unfolded response (UPR) with small molecules and natural products has recently been demonstrated to be a productive approach in pre-clinical models of OSCC and several other cancers. The aim of current study was to perform a high throughput screen (HTS) with a diverse chemical library to identify compounds that could induce CHOP, a component of the apoptotic arm of the UPR. Disulfiram (DSF, also known as Antabuse) the well-known aversion therapy used to treat chronic alcoholism emerged as a hit that could generate reactive oxygen species, activate the UPR and apoptosis and reduce proliferation in OSCC cell cultures and xenografts. A panel of murine embryonic fibroblasts null for key UPR intermediates (e.g., Chop and Atf4) was resistant to DSF suggesting that an intact UPR is a key element of the mechanism regulating the antiproliferative effects of DSF.

## 1. Introduction

The Oral Cancer Foundation (OCF) has accurately predicted the annual incidence of oral (OSCC) and oropharyngeal cancer in the United States since 2002. OCF anticipates that 2019 will see 50,000 new cases with about 10,000 deaths, substantially higher than in 2000 when the expected number was ~30,000 and had been unchanged for the previous two decades [1]. A paucity of treatments and an age shift down in high risk behaviors including alcohol and tobacco use, and the sexual comportments of young people, have likely contributed to this alarming trend [2,3,4,5]. The burden of squamous cell carcinomas of the head, neck, oral cavity and nasopharynx, and larynx is much higher in less developed countries where variations of smokeless tobacco and the chewing of betel and areca nuts are cultural norms, and outcomes are much worse [6,7,8]. 

The high morbidity and mortality associated with OSCC are bolstered by the fact that approximately 60% of patients present with late stage disease. The primary modes of treatment for OSCC remain in the purview of surgeons and radiation oncologists as the addition of chemotherapy and biological agents have provided only modest improvements in the last three decades. Promising clinical trials in 2016 and 2017 saw the introduction of immune modulating checkpoint inhibitors to the arsenal of FDA approved compounds for late stage and recurrent head and neck cancer however, these compounds are not yet approved for OSCC and there is an urgent unmet clinical need to find new approaches for patients afflicted with this devastating disease. 

Our group has previously hypothesized that targeting the unfolded protein response (UPR), to increase nuclear pools of C/ebp homologous protein (CHOP), might specifically leverage the highly secretory nature of OSCC towards its demise [9,10,11]. Every protein destined for secretion from the cell or that resides in the cell membrane is translated at the endoplasmic reticulum (ER) membrane-lumen interface and subsequently folded and post-translationally modified in the lumen. The secretory machinery involves a complex array of foldases, chaperones and tightly regulated quality control mechanisms to effectively fold proteins into their native conformation. A number of pathologic conditions (especially cancer) and pharmacologic challenges can interfere with folding and lead to ER stress, the UPR and the accumulation of CHOP.

mRNA molecules are translated into proteins with different efficiencies depending on the length and complexity of the structure of their 5’ untranslated regions. Many oncogenes have long highly unstructured 5’ UTRs that are not conveniently accessed by translation initiation factors, which keeps their translation at low levels during homeostasis. It is now clear that eukaryotic translation initiation factor 2, (eIF2α) and eIF4E are mis-regulated in a subset of acute [12,13] and chronic myelogenous leukemias, non-Hodgkin B-cell lymphoma, colon cancers [14], breast carcinomas [15], and squamous cell carcinomas of the head and neck [16,17], increasing the likelihood that quiescent oncogenes might become aberrantly expressed.

The UPR is a remedy to perturbations in the secretory pathway that disrupt protein folding, post-translational modification, or trafficking out of the ER. The UPR is orchestrated by a tripartite signaling conduit characterized by a transient halt in general protein translation and increased expression of folding-associated enzymes [18]. The ER transmembrane kinase PERK phosphorylates the alpha subunit of eIF2α to attenuate protein synthesis [19], and ER-localized activating transcription factor 6 (ATF6) is processed to yield a cytosolic fragment transcription factor that activates UPR gene expression [20]. The most conserved UPR subpathway is mediated by IRE1α [21] which initiates removal of a 26 base intron from X-box binding protein (*XBP1*) to produce a potent basic leucine zipper transcription factor that increases the expression of ER chaperones and proteins that enhance ER-associated degradation (ERAD) [22,23]. If these pathways are unable to restore homeostatic protein folding due to prolonged or robust stress, cells undergo apoptosis primarily through ATF4-mediated transcriptional induction of CHOP [24].

As CHOP is known to be a mediator of ER stress-induced apoptosis, a cell-based high throughput screen (HTS) was undertaken to identify small molecule activators [25]. We hypothesized these agents would exacerbate the UPR and induce apoptosis in cells with high basal levels of ER stress (e.g., OSCC) and produce only minimal off target toxicity to normal unstressed cells/tissues. Malignant cells are thought to be more sensitive to ER stress (than normal cells) due to, among other factors, a drastically increased need for de novo protein synthesis to support aberrant growth and increased secretion of chemokines. Additionally, many malignant cells are aneuploid, which can result in an unbalanced expression of subunits of multi-protein complexes and accumulation of unfolded proteins. 

One of the agents identified in the HTS screen was tetraethylthiuram disulfide, also known as disulfiram (DSF), that has been used safely and frequently at large oral doses for the treatment of alcoholism since 1948. DSF and various metabolites are potent inhibitors of mitochondrial and cytosolic aldehyde dehydrogenases (ALDH) [26,27]. DSF enhances the anticancer effects of cisplatin, cyclophosphamide, and paclitaxel, and radiation in vitro and protects nonmalignant tissues from the harmful effects of cisplatin in the kidney, bladder, gastrointestinal tract and bone marrow in vivo [28,29,30,31]. The ability of DSF to induce the UPR and reduce OSCC tumor burden in vivo has not been described. 

## 2. Materials and Methods

### 2.1. Cell Lines and Culture

A253 and Cal27 cells were obtained from ATCC; HN6, HN12, and HN30 cells were provided by Dr. George Yoo at Karmanos Cancer Institute, and STR-validated UMSCC-1, -14A and -23 cells were from Dr. Tom Carey at the University of Michigan. All cancer cell lines were maintained under standard tissue culture conditions in DMEM supplemented with 10% FBS and antibiotics as previously described [32]. Normal human epidermal keratinocytes from ScienCell (Carlsbad, CA, USA) were cultured per the manufacturer’s protocol with the recommended culture medium and supplements.

### 2.2. Reagents

DSF and NSC-1771 were obtained from MicroSource Discovery Inc. (Gaylordsville, CT, USA) or Acros Organics (Morris Plains, NJ, USA). 100 mM solutions were prepared in DMSO and stored at −20 °C. Approximately 60,000 small molecules and natural product extracts were screened at the University of Michigan Center for Chemical Genomics as previously described [25].

### 2.3. Carbonyl Assay

To detect oxidized proteins in lysates from OSCC cells treated with DSF, the Protein Carbonyl Colorimetric Assay Kit from Caymen (Ann Arbor, MI, USA) was used according to the manufacturer’s protocol. 

### 2.4. ^35^S. labeling and TCA Precipitation

5.5 × 10^5^ SCC-14A cells were plated in triplicate the day before treatment with 10 μM DSF or 0.5 μM thapsigargin (Tg). At harvest, cells were rinsed twice with PBS and 5 μL ^35^S-Met was added and incubated for 15 min at 37 °C. ^35^S-Met medium was removed and plates were placed on ice and washed twice with cold PBS. Whole cell lysates were prepared in 200 μL RIPA with PMSF without protease inhibitors. 5 μL of each sample in 5 mL of scintillation fluid was used to measure cpm; values were normalized to total proteins assay by the Lowry method [33].

### 2.5. Proliferation and Cell Death

Cell Titer Glo, an ATP-based luminescent proliferation assay from Promega (Madison, WI, USA), was used as a relative measure of viable cells. Trypan-blue exclusion assays were employed to determine the ratio of live:dead cells. 

### 2.6. Immunoblotting 

Whole-cell lysates were prepared in modified radioimmunoprecipitation (RIPA) buffer with PMSF and protease inhibitors. Briefly, 50–80 micrograms of cell lysate was resolved on 7.5% or 12.5% SDS-PAGE gels and electrically transferred to PVDF membranes (Bio-Rad, Hercules, CA, USA). Membranes were probed overnight at 4 °C with antibodies for caspases 3, caspase 9, GRP78/BIP, CHOP and phosphorylated eIF2α (Cell Signaling, Danvers, MA, USA); ATF4 (Santa Cruz Biotechnology, Santa Cruz, CA, USA), as previously described [34]. Following incubation with relevant secondary antibodies, membranes were incubated with SuperSignal west pico ECL-HRP substrate (Thermo Fisher Scientific, Waltham, MA, USA) to detect relative protein. GAPDH (Millipore, Billerica, MA, USA) was used to probe each stripped membrane to verify relative protein loading.

### 2.7. RT-qPCR

mRNA was harvested from DSF treated UMSCC-1 and -14A cells using Cells to CT™ (Ambion, Foster City, CA, USA) as previously described [35]. Semi-quantitative RT-PCR analysis of un-spliced and spliced *XBP1* was performed with a single human-specific primer pair ACA CGC TTG GGA ATG GAC AC and CCA TGG GAA GAT GTT CTG GG [36]. RT-qPCR was performed with the following Taqman primer/probes: *18S* (Hs99999901_s1), *CHOP/DDIT3* (Hs01090850_m1), *GADD34/PPP1R15* (Hs00169585_m1), *ATF3* (Hs00910173_m1), *ATF4* (Hs00909569_g1), *XBP1s* (Hs00231936_m1), and *GRP78/BiP/HSPA5* (Hs99999174_m1). All experiments were performed with triplicate samples and each measured at least three times; data are represented as standard error of the mean. 

### 2.8. Xenografts and Bioluminescent Imaging (BLI)

All animal studies were performed in accordance withthe Wayne State University IACUC (protocol ID 16-07-111). 10^6^ SCC23-CHOP-luc cells were injected subcutaneously in the flanks of 6–12 week old female athymic SCID mice and allowed to grow until palpable solid masses were appreciable (~3 mM in diameter). Mice were randomized to 100 mg/kg DSF or carrier (olive or almond oil) daily delivered by oral gavage. Xenograft volume was calculated using ½ (L X W^2^), where length and width are the widest. Daily oral gavage was continued until tumors exceeded 1cm^2^ or animals became moribund as per WSU IACUC protocol. Bioluminescent images of CHOP-luciferase expressing cells were captured 24 h after DSF or vehicle administration; random mice were IP injected with 100 mg/kg luciferin and imaged on a Kodak In-Vivo Extreme. 

### 2.9. Statistical Analysis

Each data set represented graphically or mentioned in the text was analyzed by a referee uninvolved with the experimental procedure. Two-way ANOVA was used to appreciate differences in proliferation between wildtype and knockout murine embryonic fibroblasts (MEF). Two-tailed tests were used to appreciate differences between treated and untreated cell cultures (i.e., gene expression, ^35^S incorporation, carbonyl formation, etc.). In each case the level of confidence was represented as follows: * *p* ≤ 0.05, ** *p* ≤ 0.01, *** *p* ≤ 0.001.

## 3. Results

**Figure 1:**
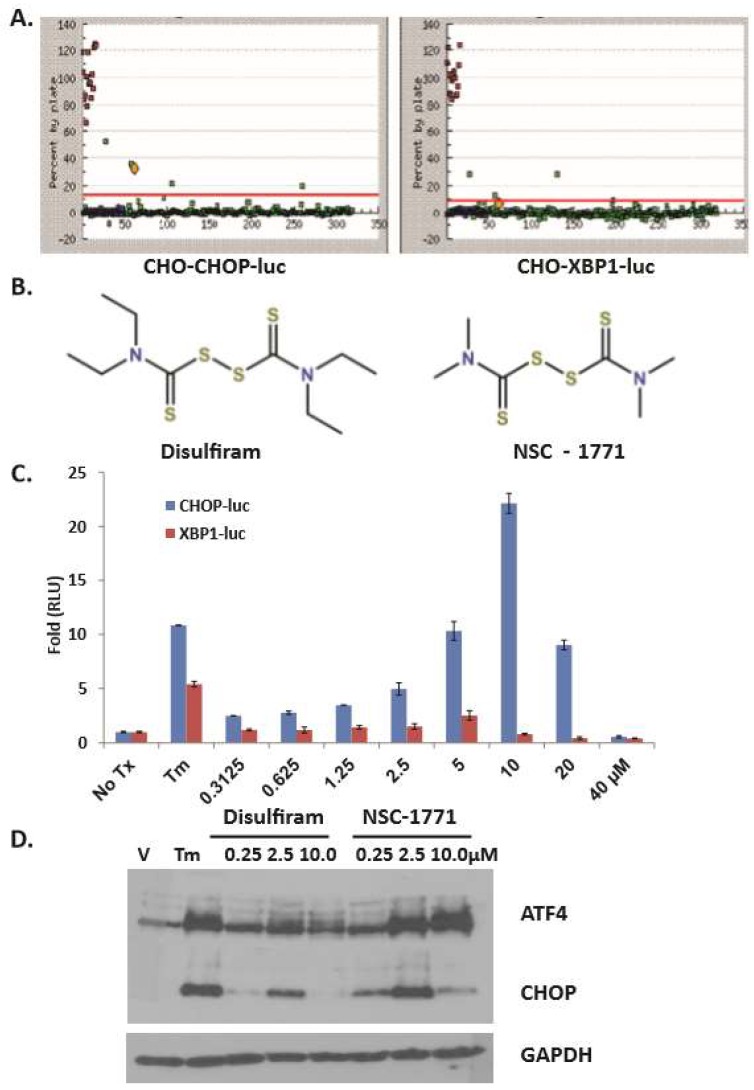
Dithiocarbamates activate *Chop* Expression. (**A**), Campaign view of a 384 well plate of CHO-Chop-luc (left) and CHO-Xbp1-luc (right) cells treated with compounds from the MicroSource Spectrum collection for 8h; green dots: high throughput screen (HTS) cmpd, blue dots: DMSO, red dots: tunicamycin (Tm); yellow dot: Disulfiram (DSF); assay Z’: CHOP = 0.84, XBP1 = 0.88. (**B**), Structure of DSF (left) and analog NSC-1771 (right). (**C**), Secondary concentration response assays with reporter cell lines; error bars represent standard deviation. (**D**), Immunoblot analysis with whole cell lysates from DSF- or NSC-1771-treated murine embryonic fibroblasts (MEF) cultures probed with polyclonal antibodies for Atf4 and Chop. A single membrane used to appreciate Atf4 and Chop was stripped and re-probed with a monoclonal antibody for GAPDH.

**HTS identification of DSF as a bona fide UPR activator.** We previously reported a productive high throughput screen (HTS) strategy used to discover small molecules and natural products that activated the apoptotic arm of the UPR [25]. The assay employed two stable CHOK1-UPR-luciferase cell lines that report individually on the PERK-eIF2α-CHOP (apoptotic) or the IRE1α-XBP1 (adaptive) arms of the UPR. CHO-CHOP-luc (Figure 1A left) and CHO-XBP1-luc cells (Figure 1A right) were treated (10 μM) with HTS compounds (green dots) from the Micosource Spectrum Collection. DSF (Figure 1B) emerged as a hit that could significantly activate the CHOP-luc but not the XBP1-luc reporter (Figure 1 yellow dot). Concentration response assays confirmed this observation (Figure 1C). DSF and the structurally related analog NSC-1771 were re-ordered as dry power stocks. Both compounds induced Atf4 and Chop expression in cultured murine embryonic fibroblasts (MEF) (Figure 1D). DSF and NSC-1771 are known copper binding dithiocarbamates; Cu-DSF conjugates have previously been shown to activate the UPR in cancer [37] however, no UPR-focused studies have been reported that employed DSF or NSC-1771 in OSCC. As NSC-1771 is used commercially as a fungicide and known to cause dyschondroplasia in poultry, it was excluded from further consideration [38].

**Figure 2:**
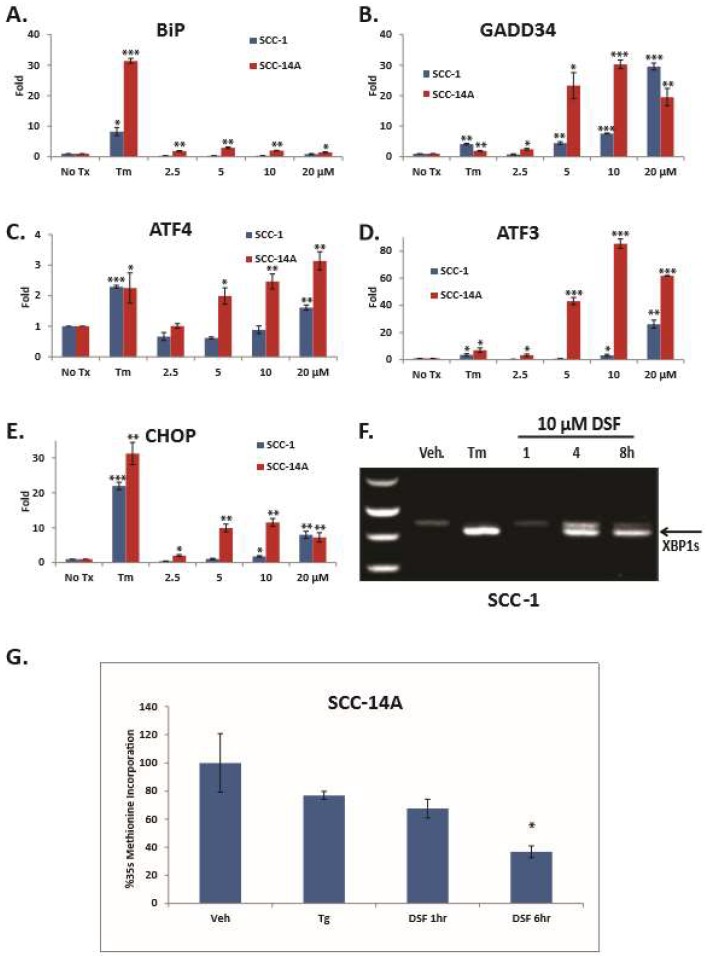
DSF activates unfolded response (UPR) gene expression and inhibits general protein translation. (**A**–**E**), RT-qPCR analysis of UPR genes; error bars represent the standard error of the mean from three experiments performed with triplicate samples. (**F**), Conventional RT-PCR to appreciate un-spliced and spliced *XPB1*. (**G**), S35 pulse-labeled and TCA precipitated whole cell lysates following 10 μM DSF treatment; error bars represent standard deviation. Two-tailed test: * *p* ≤ 0.05, ** *p* ≤ 0.01, *** *p* ≤ 0.001.

**DSF induces ER stress and the UPR in OSCC cell lines.** To elucidate the ability of DSF to activate the UPR, SCC-1 and SCC-14A cells were treated for six hours. RT-qPCR revealed dose-dependent increases of transcripts for UPR markers (Figure 2A–E). To independently confirm UPR gene expression a UPR-focused quantitative RT-PCR array was used to interrogate cDNA from DSF-treated SCC1 cells. Transcripts for *CHOP*, *ATF4*, *GADD34* and a panel of HSPs were significantly upregulated (Supplementary Figure S1). Conventional RT-PCR was used to measure *XBP1* splicing (Figure 2F). UMSCC-14A cells were then treated over a six hour period and pulse labeled with ^35^S-Met. Trichloroacetic acid (TCA) precipitation and radioanalysis confirmed 10 μM DSF could rapidly and significantly reduce protein synthesis (Figure 2G). Considered together, these data indicate that DSF induces ER stress and the UPR in OSCC.

**Figure 3:**
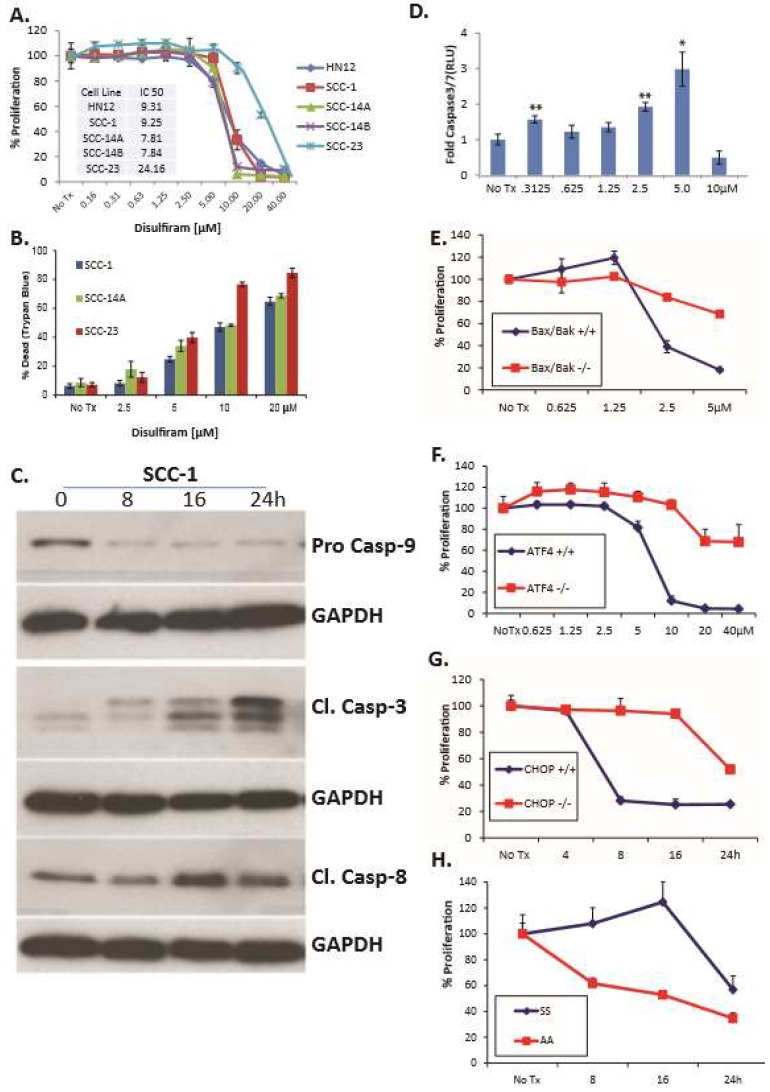
DSF induces UPR-dependent apoptosis and reduces proliferation in OSCC. (**A**), A panel of OSCC cells treated with increasing doses of DSF for 24 h evaluated with an ATP-based luminescent proliferation assay; data are representative of multiple experiments; error bars represent standard deviation. (**B**), Similarly treated cell lines evaluated after 24 h with trypan blue exclusion assay to appreciate relative levels of live and dead cells; error bars represent standard deviation. (**C**), Immunoblot analysis of caspases in whole cell lysates from SCC-1 cells treated with 10 μM DSF. (**D**), Luminescent real-time measurement of caspase3/7activation in MEFs treated for 4h; error bars represent standard deviation. Two-tailed test: * *p* ≤ 0.05, ** *p* ≤ 0.01, *** *p* ≤ 0.001. (**E**–**H**), ATP-based proliferation assays in MEF treated with 5 μM DSF. *Bax/Bak* double-null MEF; Two-way ANOVA, *p* ≤ 0.0001 for concentration and interaction, *Atf4*-null MEF; *p* < 0.0001 for concentration and interaction. *Chop*-null MEF; *p* ≤ 0.0001 for time and interaction and eIF2α Ser51-Ala mutant (A/A) MEF; *p* ≤ 0.0001 for time and interaction. Wildtype controls were littermate matched MEFs; error bars represent standard deviation.

**DSF induces OSCC apoptosis and reduces proliferation.** A panel of five OSCC cell lines was treated with DSF for 24 h; ATP-based luminescent proliferation assays revealed dose-dependent growth inhibition (Figure 3A). Trypan blue-exclusion assays determined the antiproliferative effect was substantially due to cell death (Figure 3B). Immunoblot analysis with lysates from DSF treated SCC1 cells revealed an accumulation of the active forms of caspase 3, caspase 8 and caspase 9 (Figure 3C); and a luminescent caspase 3/7 assay in MEFs plated at the same time confirmed the presence of active caspases (Figure 3D). MEFs doubly deficient for proapoptotic Bax and Bak were resistant to DSF, further implicating the intrinsic (mitochondrial-mediated) apoptotic pathway as a feature of DSF-induced cell death (Figure 3E). To examine the role of the UPR in DSF induced apoptosis, a panel of UPR-mutant MEFs and wildtype littermate control cells was employed. MEFs null for *Atf4* or *Chop* could withstand relatively high concentrations of DSF (Fig. 3 F and G), and phosphorylation resistant eIF2α Ser51Ala mutant (A/A) MEFs were much more sensitive to the antiproliferative effects of DSF than wildtype (S/S) (Figure 3H), consistent with previous reports that this mutant is intolerant to ER stress [39]. Normal human epidermal keratinocytes continued to proliferate in relatively high concentrations of DSF (IC50 56.6 μM) and *CHOP* expression was not apparent until 80 μM in concentration response assays (Supplementary Figure S2A,B). Considered together these data indicate that DSF induced apoptosis in OSCC and that activation of the UPR is upstream of (and required for) efficient apoptosis in MEFs.

**Figure 4:**
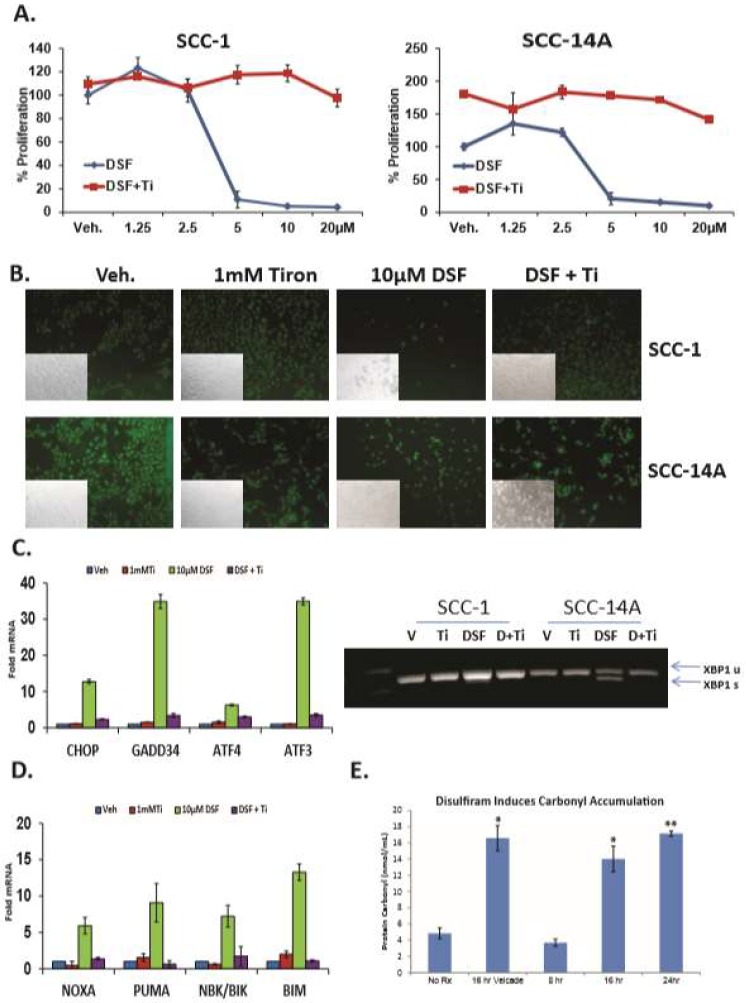
DSF-mediated reactive oxygen species ROS are required for UPR activation and leads to oxidative protein damage. (**A**), Proliferation assays with oral (OSCC) cells treated with increasing concentrations of DSF in the presence or absence of the superoxide scavenger tiron (Ti) for 24h, error bars represent standard deviation. (**B**), Identically treated cells stained with fluorescent dyes to appreciate live cells (green) and dead cells (red) after 24 h. (**C**), RT-qPCR (left) and RT-PCR (right) to appreciate UPR genes in OSCC cells treated with 10 μM DSF +/− tiron for 6 h. (**D**), RT-qPCR analysis of the same cDNA libraries as in (**C**) for apoptotic transcripts. (**E**), Analysis of carbonyl accumulation as a marker of protein damage in 10 μM DSF treated OSCC cells (8–24 h), as indicated; error bars represent standard deviation, two-tailed test: * *p* ≤ 0.05, ** *p* ≤ 0.01, *** *p* ≤ 0.001.

**DSF induced reactive oxygen species (ROS) are required for UPR induction in OSCC.** DSF is well-known and potent redox modulator, and ROS generation is a well-characterized stimulus of ER stress. To more precisely elucidate the mechanism by which DSF induced ER stress and the UPR, OSCC cells were treated with 10 μM DSF for 24 h in the presence of the potent superoxide-specific scavenger tiron (Ti). ATP-based luminescent proliferation assays (Figure 4A) and a fluorescent live/dead assay (Figure 4B) revealed that 1 mM tiron protected both cell lines over a 24 hour period. The same concentration of tiron almost completely blocked the ability of DSF to activate UPR (Figure 4C) and apoptotic (Figure 4D) biomarker genes in SCC-1 cells after eight hours. Tiron treatment also abolished the ability of DSF to induce the oxidative stress genes *HO1* and *MnSOD* (Supplementary Figure S3). High levels of ROS are known to cause damage to DNA, protein and lipid membranes and lead to apoptosis. Measurement of the carbonyl content of proteins subject to stress is a common and reliable proxy for protein oxidation [40]. Carbonyl assays performed with lysates from SCC-1 cells treated with ROS-inducing bortezomib [32] and 10 μM DSF revealed significantly increased levels of oxidized proteins at 16 and 24 h (Figure 4E). Considered together, these data reveal that treatment of OSCC cultures with DSF led to protein damaging ROS that activated the UPR and apoptosis.

**Figure 5:**
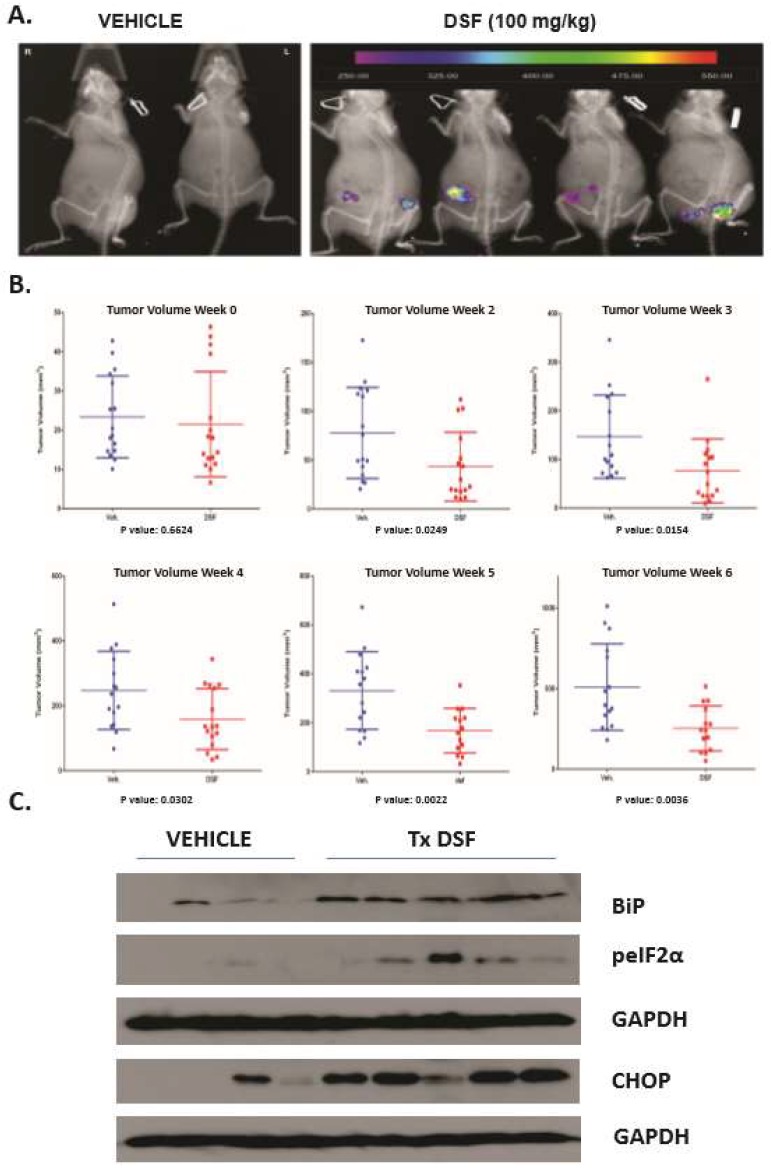
DSF activates UPR in OSCC xenografts and reduces tumor burden. (**A**), Bioluminescent imaging (BLI) to appreciate CHOP-luciferase activity in mice bearing 3–5 mM tumors. (**B**), Volumetric (caliper) evaluation of tumors in 8 mice bearing bilateral xenografts and treated either with almond oil carrier or compounding pharmacy prepared DSF over 6 weeks. (**C**), Immunoblot analysis of UPR markers in whole tumor lysates prepared from xenografts 8 h after final dose of DSF or vehicle, blots were stripped and re-probed for monoclonal antibodies against human GAPDH.

**DSF induces UPR in OSCC xenografts and reduces tumor burden.** DSF was reported to have anti-tumor effects in several preclinical rodent xenograft cancer models. To elucidate whether DSF could induce the UPR and reduce tumor burden in OSCC, flank xenografts where generated with SCC-23-CHOP-luciferase cells in female SCID mice. 5–7 days after injection mice bearing ~3 mM palpable masses of were treated with 100 mg/kg DSF or equal volume olive oil carrier by oral gavage. Bioluminescent imaging (BLI) revealed CHOP-luc activity reporter was apparent in DSF treated mice after 24 h (Figure 5A). BLI was not useful to measure CHOP-luc expression after the control tumors grew to ~>5 mM due to increased basal levels of UPR activation and increased basal CHOP-luc expression (not shown). Over the course of 30 days DSF treated mice displayed significantly smaller tumor burdens than controls (Figure 5B). Mid-term and 30 day computer aided tomography (CAT) analysis performed by two calibrated blinded pathologist reviewers confirmed the caliper-derived volumetric analysis (Supplementary Figure S4A,B). At the termination of the experiment whole tumor lysates were prepared and interrogated for UPR markers. DSF treated xenografts had increased levels of GRP78/BiP, CHOP and phosphorylated eIF2α indicating increased UPR activity (Figure 5C). A replicate experiment using another preparation pharmaceutical grade DSF (in almond oil carrier) produced the same reduction of SCC-23-CHOP-luc tumor burden (Supplementary Figure S5A,B). These data indicate that DSF enforced UPR activation prevented tumor growth in an early-stage murine xenograft model of OSCC.

## 4. Discussion

The noxious reaction occurring upon the combination of ethanol and DSF was reported by E. E. Williams while working as the plant physician at a chemical company producing tetramethylthiuram disulfide and tetramethlythiuram monosulfide for vulcanizing (hardening) agents used in the production of rubber [41]. Williams prognosticated the potential of DSF to be used as a treatment for chronic alcoholism, noting that in the absence of ethanol the compound was quite benign but that the mechanism of action was unknown. In back to back articles in the same issue of the Lancet in 1948 Hald and Jacobsen [42] and Martensen-Larsen [43] reported results from clinical trials that confirmed Williams’ alcohol aversion therapy forecast. During the 1950s a serendipitous discovery by Hald and Jacobsen revealed that irreversible inhibition of aldehyde dehydrogenase (ALDH) was a key mechanistic feature to their previous observation [26]. During alcohol metabolism acetylaldehyde is reduced to benign acetate before leaving the body as carbon dioxide and water. Inhibition of ALDH produces toxic levels of acetylaldehyde that are responsible for the characteristic flushing and prodigious nausea and vomiting that occur when DSF and ethanol are combined. As DSF is a well-characterized FDA-approved drug with few side effects that has been studied in myriad malignancies; we tested the hypothesis that DSF could induce ER stress, and UPR-mediated apoptosis in OSCC. 

The anticancer effects of DSF have been studied in many pre-clinical cancer models including brain [44], glioblastoma [45], liver [46,47], melanoma [48], prostate [49], pancreas [50], leukemia [51,52], breast [53,54] and malignant pleural mesothelioma [55]. To our knowledge there has been only one preclinical study that employed DSF in an OSCC model [56]. Jivan and colleagues hypothesized in that study that the increased activity of protein degradation/turnover pathway required by cancers might predispose them to the antiproliferative effects of DSF, and demonstrated that the diabetic drug metformin could enhance DSF- and Cu-DSF mediated cell death in vitro, possibly due to synergy of ROS. The broad range of cancers that are sensitive to DSF suggests that DSF might be targeting a common feature of malignancy rather that a specific signaling intermediate or pathway. 

The cancer stem cell (CSC) hypothesis is rooted in the idea that tumor proliferation, metastasis, resistance to therapy and recurrence are driven by a small subset of progenitor cells. CSC’s are characterized by the ability to reproduce progenitor cells (self-renewal) and to divide into daughter cells that populate tumors [57]. Although not all cancers grow in a fashion consistent with this hypothesis, it is generally well-recognized that squamous cell cancers of the oral cavity and head and neck do [58]. OSCC stem cells are most commonly identified by the expression of ALDH [59] and CD44 [60], although other markers have been identified (See Curtarelli et al., for a detailed systematic review [61]). Importantly, ALDH positive head and neck cancer cells are more tumorigenic than ALDH negative cells [59,62]. Furthermore, ALDH inhibition with siRNA or the theophylline-based ALDH1A1 inhibitor NCT-501 reduced self-renewal of Cal27 (tongue SCC) in spheroid assays and partially overcame cisplatin resistance [63]. Currently, it is not understood how DSF might target OSCC stem cell populations, or whether ALDH inhibition might modulate ER stress. Ongoing studies are focused in this regard.

The aberrant proliferation and secretory nature of malignant cells are dependent on the uninterrupted manufacture of de novo peptides. When protein folding demands outstrip the ability of the ER to fold and post-translationally modify new polypeptides the UPR is activated to restore homeostasis or eradicate the cell. Increased expression of ER stress markers and eukaryotic translation initiation factors in cancers of the head and neck, oral cavity and thyroid have been widely reported [16,17,64,65]. We and others have hypothesized that high basal levels of UPR-regulated proteins (e.g., GRP78/BiP) prime OSCC cells to get shuttled toward an apoptotic fate in the face chemically (drug) induced stress [56,65]. GRP78/BiP is also highly expressed in breast, prostate, melanoma and lung cancer [66]. The current study demonstrates the ability of DSF to exploit this feature by providing an overwhelming ER stressor that activates the UPR and reduces OSCC proliferation in vitro and in xenografts implanted into the flanks of SCID mice. MEFs null for *Chop* or *Atf4* required much higher concentrations of DSF to reduce proliferation than wildtype littermate controls in a time and concentration dependent manner, indicating an intact PERK arm of the UPR is required for efficient DSF-mediated apoptosis. 

Malignant cells generally have higher ROS activity than non-malignant cells [67] and it was hypothesized that increasing ROS pressure pharmacologically might exhaust the cellular antioxidant capacity and push cancer toward apoptosis [68]. Consistent with this notion, the ability of DSF to induce the UPR and reduce proliferation was almost entirely abolished by a superoxide scavenger administered to cultures simultaneously. Superoxide (O_2_^−^) formation occurs at the outer mitochondrial membrane, on both sides of the inner mitochondrial membrane and in the matrix, and can traverse to the cytosol freely when a mitochondrion is damaged or through voltage-dependent anion channels is response to various stimuli [69]. Superoxide is not a particularly robust oxidant, it is however, a precursor of many other (most) reactive oxygen species, and it most certainly becomes involved in the promulgation of oxidative chain reactions that can damage cellular organelles. Although in many instances the outer ER and mitochondrial membranes are in close proximity, the mechanism by which superoxide induces ER stress, other than perhaps, by directly damaging nascent polypeptides, is not known [70]. This observation is consistent with our previous finding that ER stress and cell death in OSCC cell lines treated with bortezomib was dependent on ROS generation [32].

Considering the HTS reporter data (Figure 1), where CHOP was preferentially activated, it was somewhat surprising that DSF induced XBP1 splicing in OSCC cells. This observation was very likely the result of the kinetics of the activation of the reporters. The expression of luciferase in the CHOP reporter was driven by ~8 kb of the murine *Chop* promoter, whereas expression of luciferase in the *Xbp1* reporter relied on the splicing of the 26 base intron and recombination of the firefly luciferase mRNA. *XBP1* spicing is currently regarded as a hallmark of ER stress, as IRE1α is the only known exoribonuclease able to remove the 26 base intron that holds *XBP1* in an inactive state. It is possible that the ability of DSF to splice XBP1 might contribute to the cancer cell selectivity. Tumor-adjacent normal cells with no or low basal UPR activation might be able to mount an effective adaptive UPR, overcome the stress, and return to homeostasis. Malignant cells on the other hand, become overwhelmed by the increased stress, and are shunted toward UPR (CHOP)-mediated apoptosis.

The successful use of DSF in preclinical models of cancer has been most encouraging when: (i) used in combination (complexed or supplemented) with divalent cations such as Zn^2+^ or Cu^2+^; and (ii) in early stage preclinical models (i.e., administered when the tumor burden was ≤5 mm^3^). An effective approach for the use of DSF in OSCC may be to treat patients with long term metronomic dosing after curative or salvage surgery in combination with a divalent cation supplement (i.e., Zn^2+^ or Cu^2+^). Since chronic alcohol consumption is a feature of many head and neck cancers DSF might provide an additional advantage to OSCC patients. With a five-year survival rate as low as 20% for patients with distant metastases from the floor of mouth, OSCC prognoses are stagnated amongst the worst of any cancer. The anti-alcohol and anti-tumor effects of DSF might provide a much needed boost in quality of life and survival.

## Supplementary Materials

The following are available online at https://www.mdpi.com/2077-0383/8/5/611/s1, Figure S1: DSF induced the expression of apoptosis genes in OSCC, Figure S2: Normal (non-malignant) epidermal keratinocytes were resistant to DSF and the induction of ER stress, Figure S3: Superoxide scavenger prevented the expression of DSF-induced antioxidant response genes, Figure S4: CT evaluation of tumor volume corroborated caliper-determined (calculated) tumor burden, Figure S5: Independent confirmation of in vivo xenograft data.
