# Supplementary material for: Disulfiram (Antabuse) Activates ROS-Dependent ER Stress and Apoptosis in Oral Cavity Squamous Cell Carcinoma
Source: J Clin Med. 2019 May 6;8(5):611. doi: 10.3390/jcm8050611 (PMC6571807; doi:10.3390/jcm8050611)
Supplement: Supplementary file 1 [file jcm-08-00611-s001.pdf]

Visualization of log2(Fold Change)

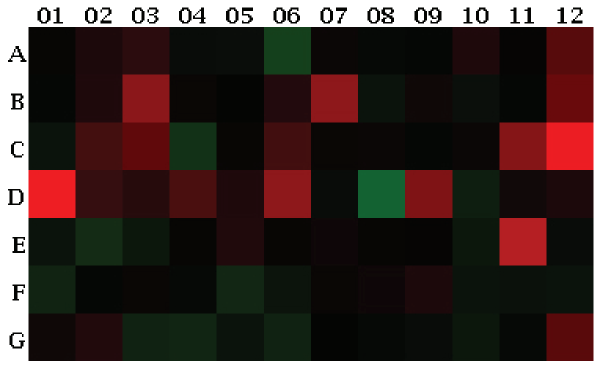

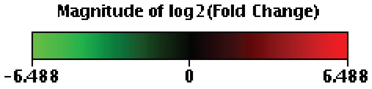

Layout	01	02	03	04	05	06	07	08	09	10	11	12
A	AMFR **1.06**	ARMET **1.70**	ATF4 **2.34**	ATF6 **-1.13**	ATF6B **-1.17**	ATXN3 **-3.14**	BAX **1.20**	CALR **-1.08**	CANX **-1.02**	CCT4 **1.76**	CCT7 **1.03**	CEBPB **4.88**
B	CREB3 **-1.03**	CREB3L3 **1.77**	DDIT3 **12.27**	DERL1 **1.14**	DERL2 **-1.01**	DNAJB2 **1.94**	DNAJB9 **12.53**	DNAJC10 **-1.26**	DNAJC3 **1.29**	DNAJC4 **-1.18**	EDEM1 **-1.02**	EDEM3 **6.67**
C	eIF2A **-1.29**	EIF2AK3 **3.73**	ERN1 **5.61**	ERN2 **-2.40**	ERO1L **1.09**	ERO1LB **3.60**	ERP44 **1.14**	FBXO6 **1.19**	GANAB **-1.05**	GANC **1.21**	HERPUD1 **10.91**	HSPA1B **67.07**
D	HSPA1L **89.73**	HSPA2 **2.84**	HSPA4 **2.10**	HSPA4L **4.08**	HSPA5 **1.79**	HSPH1 **12.62**	HTRA2 **-1.10**	HTRA4 **-5.67**	INSIG1 **9.50**	INSIG2 **-1.65**	MAPK10 **1.34**	MAPK8 **1.71**
E	MAPK9 **-1.28**	MBTPS1 **-2.19**	MBTPS2 **-1.40**	NPLOC4 **1.08**	NUCB1 **1.81**	OS9 **1.10**	PDIA3 **1.24**	PFDN2 **1.07**	PFDN5 **1.05**	PPIA **-1.44**	PPP1R15A **25.95**	PRKCSH **-1.10**
F	RNF139 **-1.79**	RNF5 **-1.03**	RPN1 **1.15**	SCAP **-1.07**	SEC62 **-1.96**	SEC63 **-1.34**	SEL1L **1.16**	SELS **1.23**	SERP1 **1.73**	SIL1 **-1.27**	SREBF1 **-1.23**	SREBF2 **-1.29**
G	SYVN1 **1.27**	TCP1 **1.89**	TOR1A **-1.74**	UBE2G2 **-1.88**	UBE2J2 **-1.28**	UBXN4 **-1.74**	UFD1L **1.01**	UGCGL1 **-1.09**	UGCGL2 **-1.12**	USP14 **-1.38**	VCP **-1.07**	XBP1 **5.02**

A.

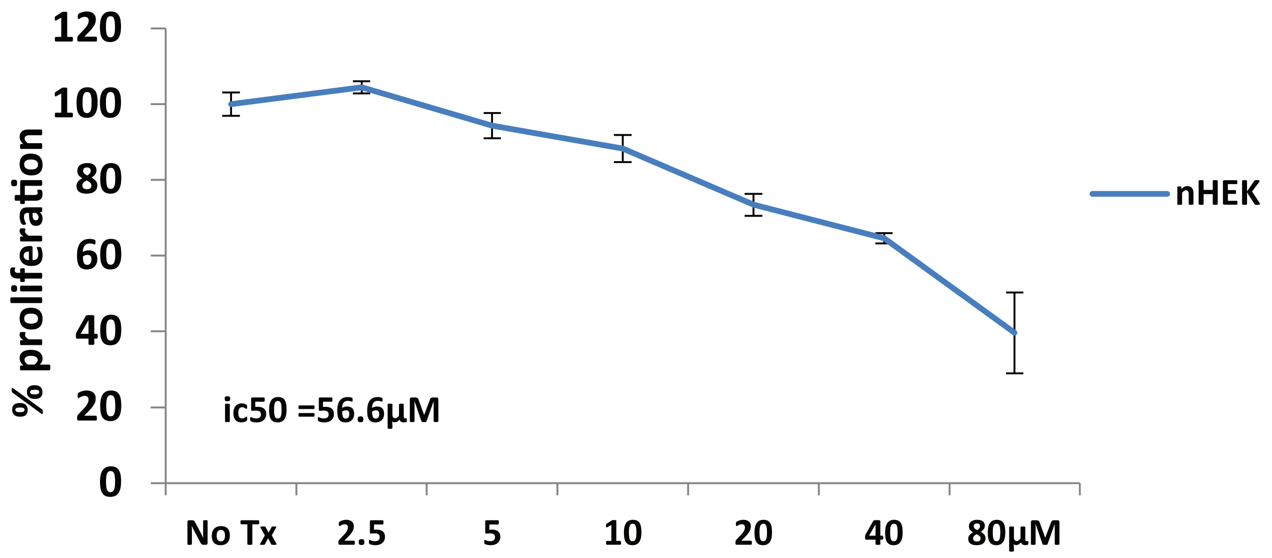
120
100
80
60
40
20
0
% proliferation
ic50 =56.6µM
No Tx
2.5
5
10
20
40
80µM
nHEK

B.

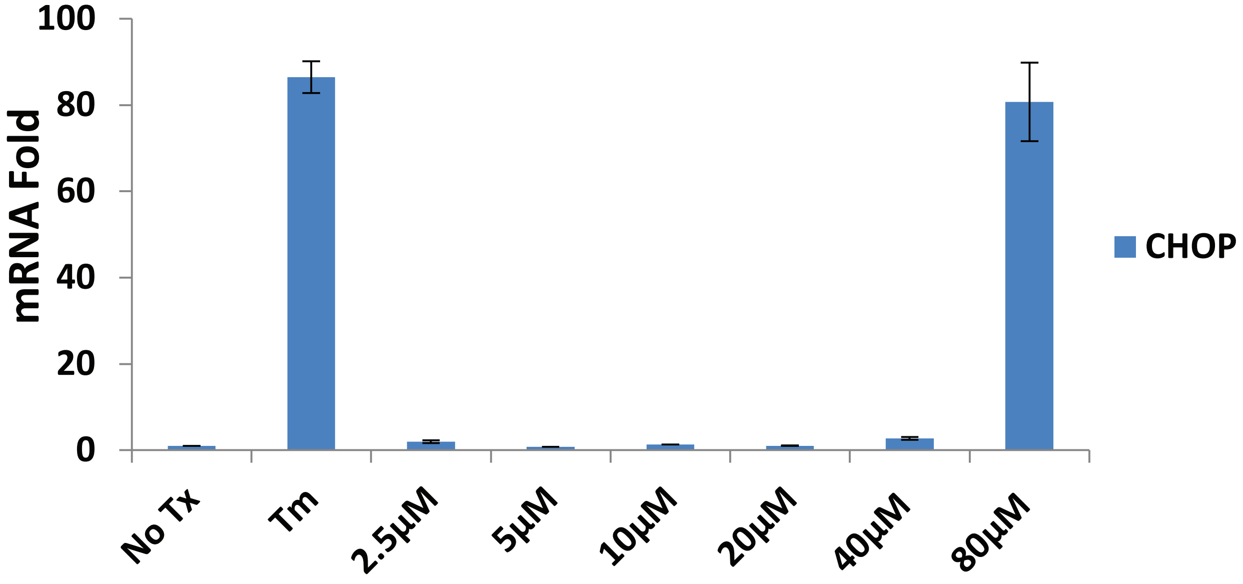
100
80
60
40
20
0
mRNA Fold
No Tx
Tm
2.5µM
5µM
10µM
20µM
40µM
80µM
CHOP

A.

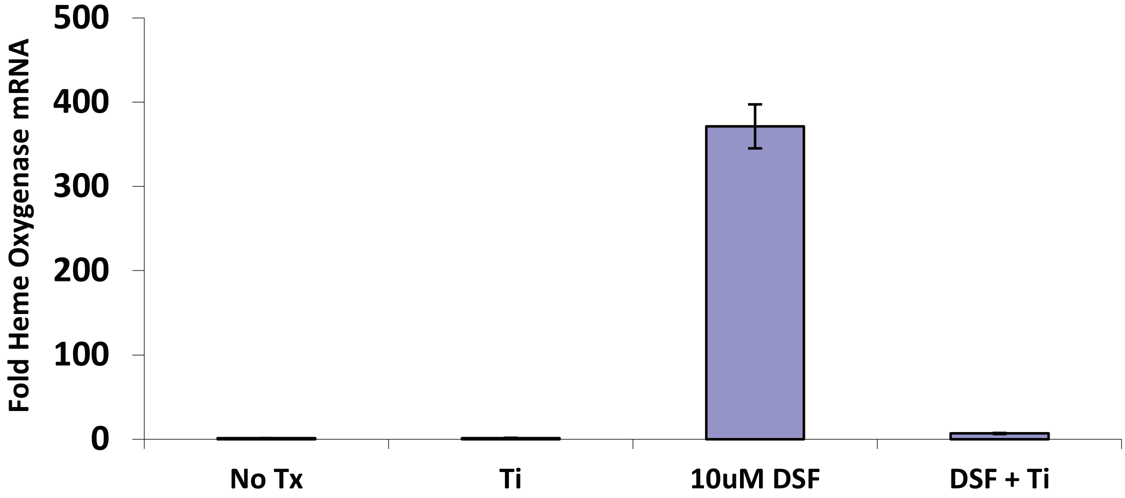
500
400
300
200
100
0
Fold Heme Oxygenase mRNA
No Tx
Ti
10uM DSF
DSF + Ti

B.

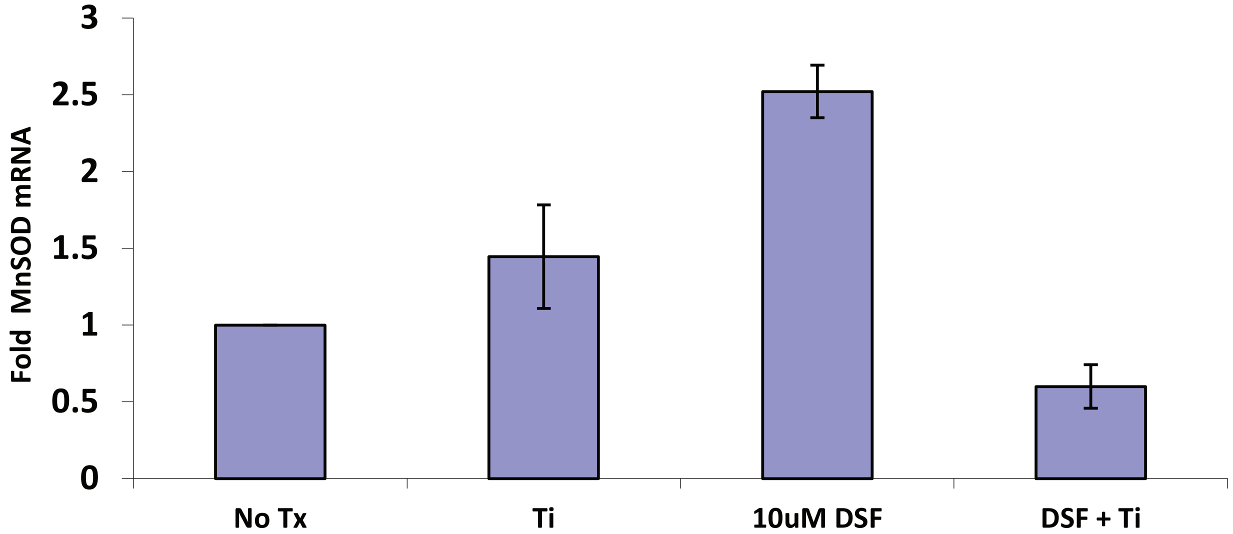
3
2.5
2
1.5
1
0.5
0
Fold MnSOD mRNA
No Tx
Ti
10uM DSF
DSF + Ti

A.

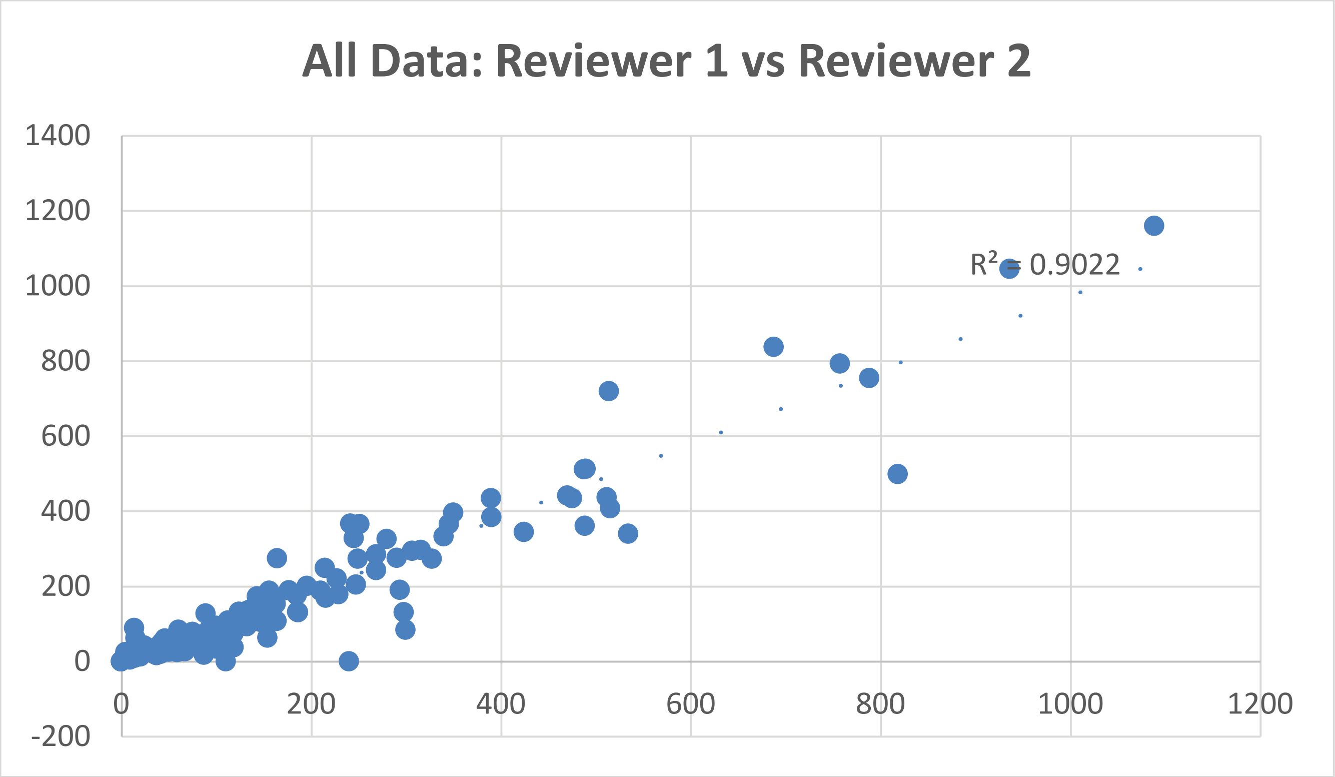
All Data: Reviewer 1 vs Reviewer 2
1400
1200
1000
800
600
400
200
0
-200
0
200
400
600
800
1000
1200
R² = 0.9022

B.

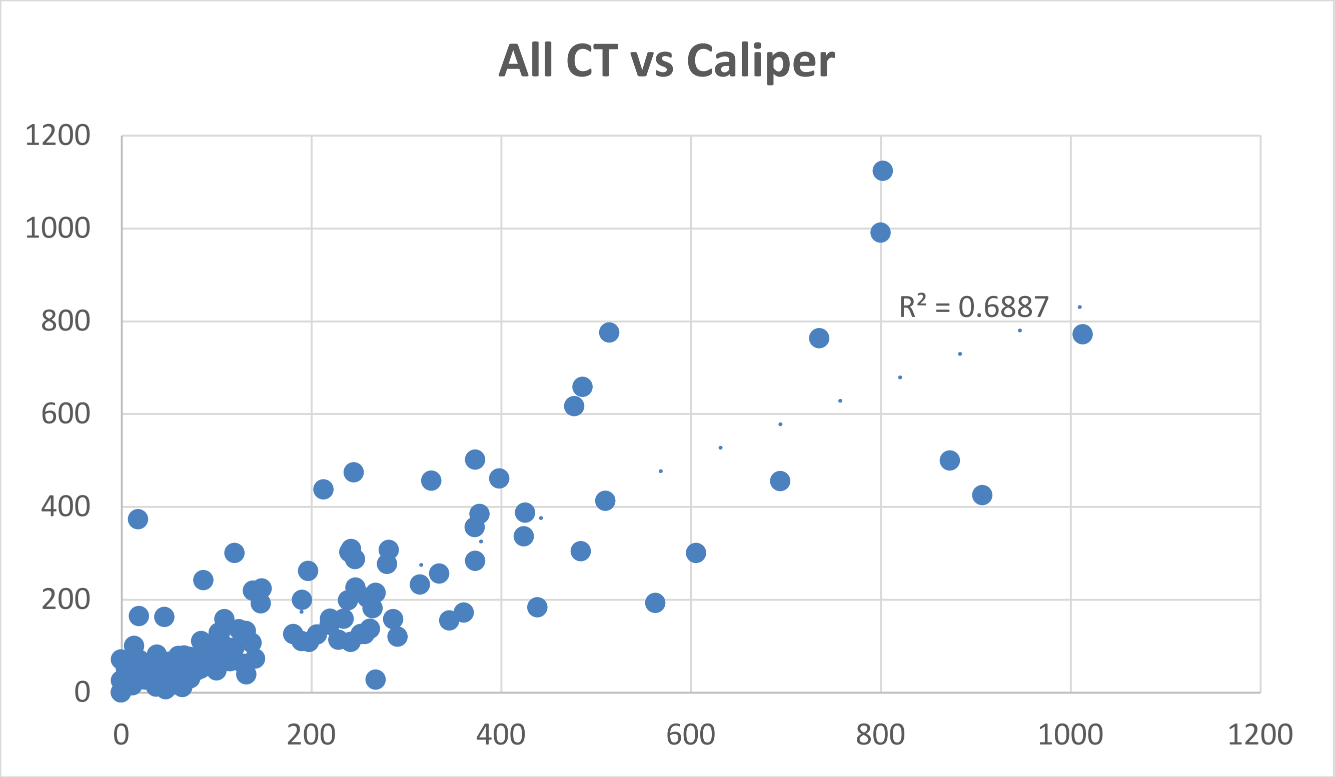
All CT vs Caliper
1200
1000
800
600
400
200
0
0
200
400
600
800
1000
1200
R² = 0.6887

A.

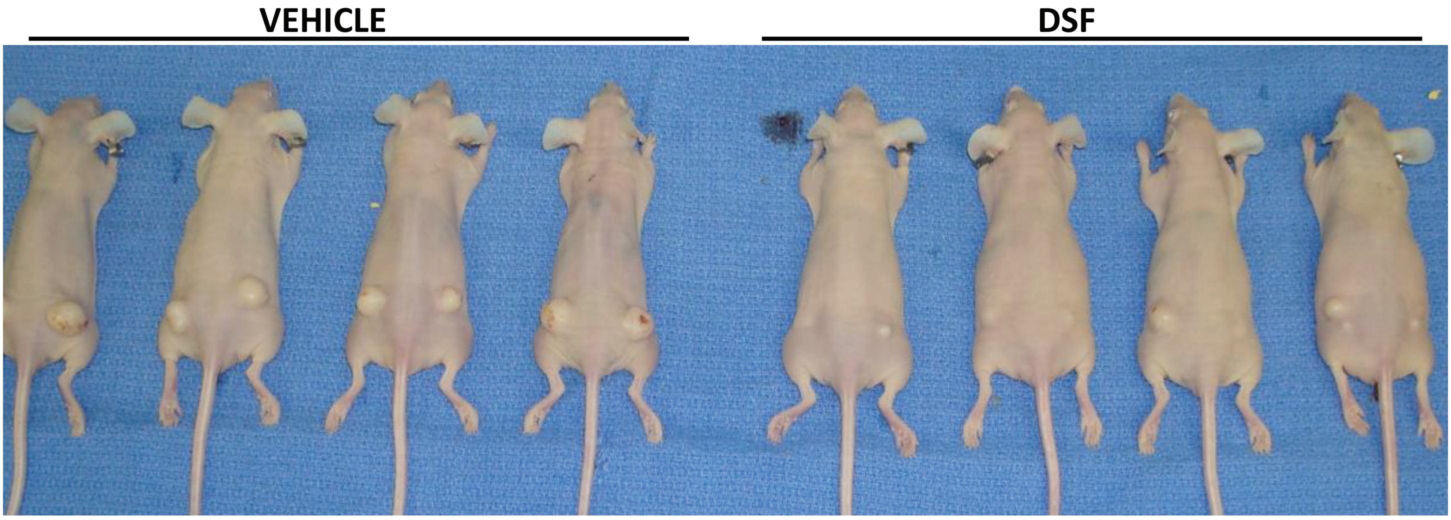
VEHICLE
DSF

B.

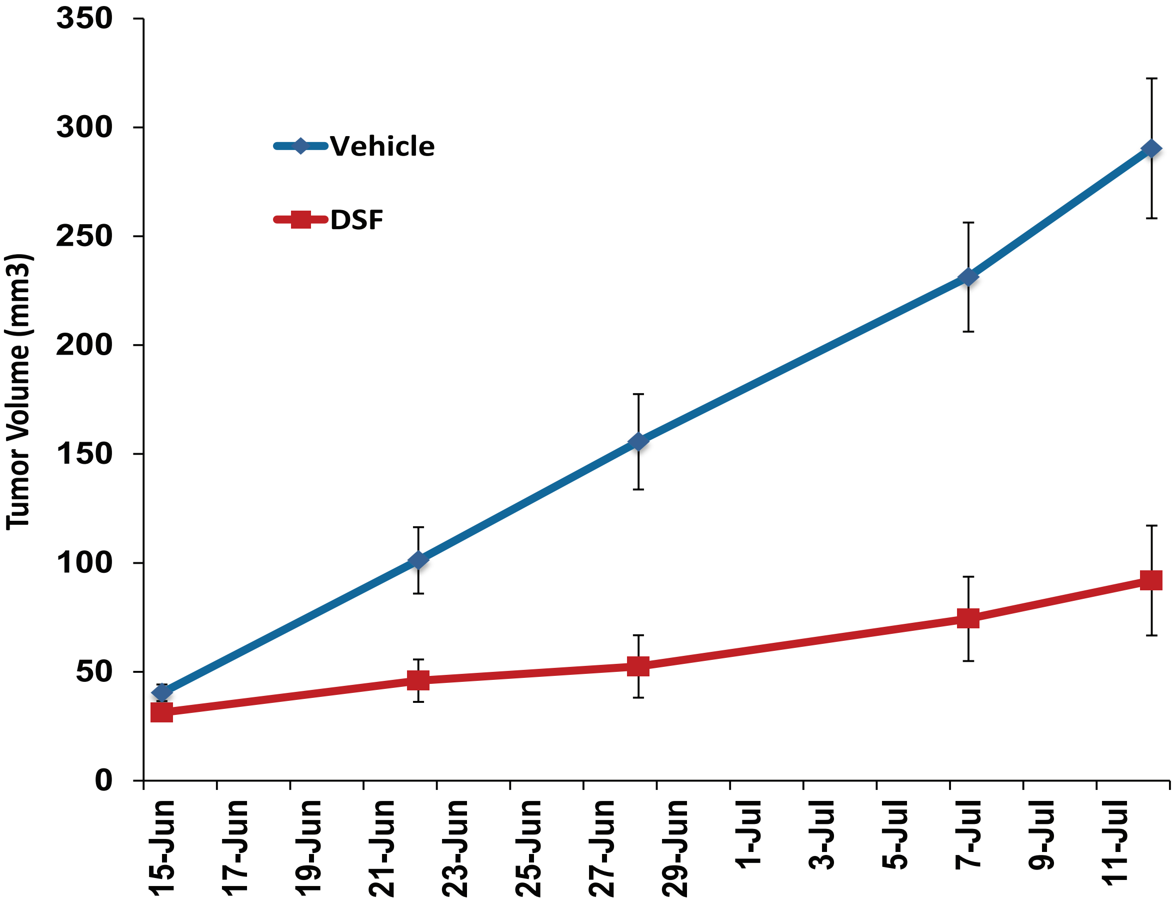
Tumor Volume (mm3)
350
300
250
200
150
100
50
0
Vehicle
DSF
15-Jun
17-Jun
19-Jun
21-Jun
23-Jun
25-Jun
27-Jun
29-Jun
1-Jul
3-Jul
5-Jul
7-Jul
9-Jul
11-Jul
